# Preparation of different thin film catalysts by direct current magnetron sputtering for hydrogen generation

**DOI:** 10.3906/kim-2003-42

**Published:** 2020-10-26

**Authors:** Gamze BOZKURT, Abdulkadir ÖZER, Ayşe BAYRAKÇEKEN YURTCAN

**Affiliations:** 1 Project Coordination Implementation and Research Center, Erzurum Technical University, Erzurum Turkey; 2 Chemical Engineering Department, Faculty of Engineering, Atatürk University, Erzurum Turkey; 3 Nanoscience and Nanoengineering Department, Graduate School of Natural and Applied Sciences, Atatürk University, Erzurum Turkey

**Keywords:** Direct current magnetron sputtering, hydrogen generation, sodium borohydride, catalyst

## Abstract

In this study, thin films of Co, Ni, Pd, and Pt were prepared on Co _3_ O _4_ support material in pellet form using the direct current (DC) magnetron sputtering method for use as catalysts for hydrogen generation from NaBH _4_ .Characterization of the catalysts was carried out using X-ray diffraction (XRD), scanning electronic microscopy (SEM), and X-ray photoelectron spectroscopy (XPS). According to cross-sectional SEM images, catalyst thicknesses were observed in the range of approximately 115.3–495.8 nm. The particle sizes were approximately 25.0, 21.4, 33.9, and 9.5 nm for Ni-Co _3_ O _4_ , Co-Co _3_ O _4_ , Pd-Co _3_ O _4_ , and Pt-Co _3_ O _4_ catalysts, respectively. The increase in NaOH initial concentration provides an increase in the rate of hydrogen generation for Co, Ni, and Pd catalysts. A maximum hydrogen generation rate of 1653 mL/g _cat_ .min was obtained for the Pt-Co _3_ O _4_ catalyst.

## 1. Introduction

The reduction of greenhouse gas emissions worldwide and the use of alternative fuels in transportation have become a forced option. According to recent research, hydrogen is an innovative fuel option for the automotive field and could replace conventional petroleum-derived liquid mixtures in passenger cars over time. However, the generation and storage of hydrogen are important issues arising from the use of hydrogen energy. Compared to physical hydrogen storage methods, chemical hydrides have superior properties for hydrogen generation. Sodium borohydride (NaBH _4_ ), which is a hydrogen storage material suitable for hydrogen generation, is the most remarkable chemical hydride due to its high hydrogen content and adjustable hydrogen release properties [1–11]. In the alkaline solution of NaBH _4_ the catalysts act as an on/off switch to provide hydrogen release [2]. This situation enables hydrogen production at the desired time. The catalytic hydrolysis reaction of NaBH _4_ is as follows:

(1)NaBH4+(2+x)H2O→catalystNaBO2.xH2O+4H2+heat

A wide variety of catalysts are used for the hydrolysis of NaBH _4_ . Supported thin film catalysts are more easily recoverable than powder catalysts, and they do not aggregate [12]. Various methods enabling effective thin film catalysts such as pulsed laser deposition (PLD), electroplating, electroless plating, induced chemical reduction, and dip coating are used to obtain supported catalysts [13–17]. In addition to these methods, the direct current (DC) magnetron sputtering method can be used for thin film catalyst production. Because of its homogeneous wide area coating, good reproducibility, and high deposition rate, the DC magnetron sputtering method is the most attractive for industrial development [18]. The catalysts prepared by the sputtering method are deposited with precise control onto the support materials as thin, compact catalytic films, and because this low-cost method does not require precursors, the emission of toxic by-products is avoided. Film composition, structure, and morphology can be changed by varying sputtering parameters such as power, inert or reactive gas flow, partial pressure, and distance between the target and surface. A DC sputtering system is used for the coating of conductive materials, while a radio frequency (RF) sputtering system is used for nonconductive materials. When the uppermost layer needs to be active for catalysts, it is unnecessary for the metal to penetrate deeply into the substrate, and catalysts can be prepared more easily by DC sputtering[12,19]. Furthermore, DC sputtering is the cheapest method because DC power supplies are simpler to manufacture than those used in RF. In the magnetron process, in addition to an electrical field for acceleration of ionized argon atoms, a magnetic field is applied perpendicular to this field. By means of the magnetic field, electrons move along the helical orbit and, thus, increase the ion concentration on the target [20].

Very few studies have reported on catalysts for hydrolysis of NaBH _4_ prepared by the sputtering method. In a study by Arzac et al., a cobalt catalyst was prepared on nickel foam by a magnetron sputtering method. They compared the hydrogen generation rates of catalysts having different film thicknesses and coated for different durations for sodium borohydride and ammonia boron hydrolysis. They reported that the highest activity for hydrogen generation from sodium borohydride was obtained from the catalyst coated for 4 h [12]. In addition, Co-based thin film catalysts are generally prepared by the different coating methods mentioned above. Therefore, the preparation of different thin film catalysts using the sputtering method for the hydrolysis reaction of NaBH _4_ is an important working area.

In this study, Co _3_ O _4_ synthesized in powder form was pelletized and coated separately with Ni, Co, Pd, and Pt metals via a DC magnetron sputtering technique applied for 20 min. The prepared catalysts were characterized by XRD, XPS, and SEM-EDS techniques. Afterwards, hydrogen generation and measurement experiments were carried out with a system designed by our group [21].

## 2. Experimental details

### 2.1. Synthesis of support material

Cobalt (II,III) oxide (Co _3_ O _4_ ) powder support material was prepared by a chemical method as previously reported [22]. The Co _3_ O _4_ synthesized in powder form was then pelletized by applying 10 tons of pressure with a manual press. The diameter and the thickness of the pellets were 13 mm and 0.2 mm, respectively. Pellets were then coated with Ni, Co, Pd, and Pt using the DC magnetron sputtering method.

### 2.2. Catalyst preparation with coating deposition

The catalysts were coated using a DC magnetron sputtering system (GSL-1100X-SPC-16M), and the conditions of the coating are given in Table 1. The distance between the substrate and the target was 40 mm, and targets with a diameter of 50.8 mm (Evochem and Quorum technologies, Ontario, Canada; 99.95% pure, 0.1–3mm thick) were used for sputtering. A mass flow controller was used to generate Ar gas flow into the chamber. The coating pressure of the vacuum level was maintained at 2.0–4.0×10 ^-2^ Mbar, and current of 20 mA was applied for 20 min which led to the formation of plasma.

**Table 1:** The coating condition of DC magnetron sputtering.

Parameters	Coating Conditions
Equipment	DC Sputtering
Target	Ni, Co, Pd, Pt (99.99%)
Base pressure	10 ^-2^ mbar
Working pressure	4x10 ^-2^ mbar
Gas	Argon
Deposition time	20 min
Power supply	AC 110V 60Hz
Applied current	20 mA

### 2.3. Characterization

The preparedcatalysts were examined by X-raydiffraction (XRD) using a PANalytical Empyrean X-ray diffractometer. The surface and cross-sectional morphology were examined by Quanta FEG 250 scanning electron microscope (SEM), and elemental composition of the coatings was determined by energy-dispersive X-ray spectrometry (EDS).The surface electronic states of the coated Ni, Co, Pd, and Pt metals were analysed by X-ray photoelectron spectroscopy (XPS) using the Specs-Flex X-ray photoelectron spectrometer.

### 2.4. Measurement of hydrogen generation rate

The activities of the catalysts were evaluated using a system designed by our group, as previously reported [21–22]. In all experiments, the effects of NaOH (99.99% pure) concentrations were investigated by stabilizing 10 wt% NaBH _4_ (98% pure) solution with different initial concentrations of NaOH(1, 10 wt%) at 25 °C.

## 3. Results and discussion

### 3.1. Characterization of the catalysts

**Figure 1:**
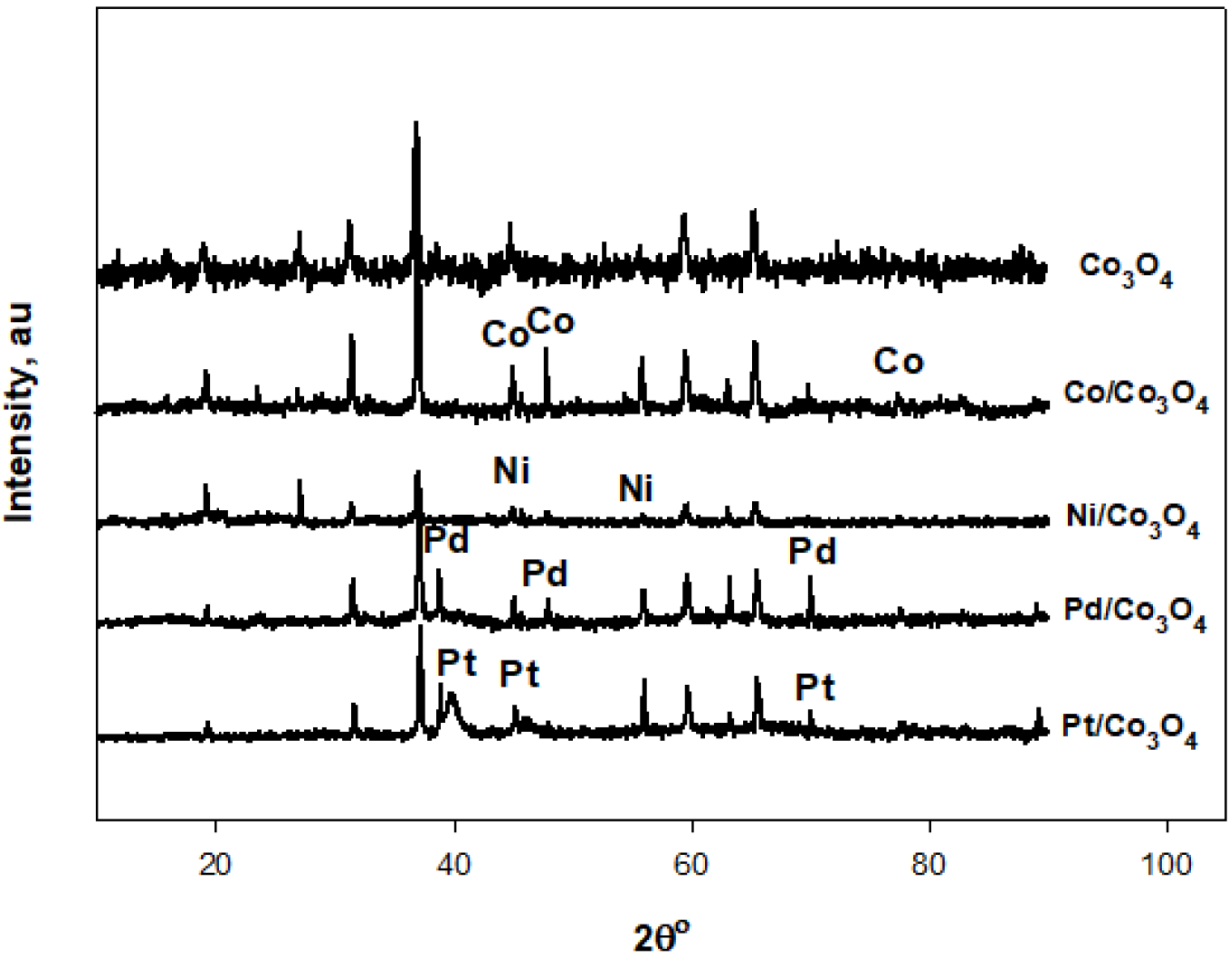
XRD patterns of the support material and catalysts.

The XRD patterns of the Co-Co _3_ O _4_ , Ni-Co _3_ O _4_ ,Pd-Co _3_ O _4_ , and Pt-Co _3_ O _4_ catalysts, which were compared with the diffraction pattern of Co _3_ O _4_ , are shown in Figure 1. According to the XRD results, Co _3_ O _4_ with a polycrystalline cubic structure was obtained. Characteristic peaks of Ni corresponding to (111) and (200) planes for 2ϴ values of 44.5 and 55.8° may overlap with the (400) and (422) planes of Co _3_ O _4_ , respectively; (002) and (101) plane peaks were observed for Co. Three diffraction peaks corresponding to the (111), (200), and (220) planes for 2ϴ values of 40.4, 46.9, and 68.6° were observed for Pd. In addition, the three peaks detected for Pt were assigned to diffraction from the (111), (200), and (220) planes for 2ϴ values of 39.6, 45.4, and 70°, respectively. The grain sizes of the prepared catalysts were calculated from the XRD data using the Scherrer equation [23]. The grain sizes were approximately 25.0, 21.4, 33.9, and 9.5 nm for Ni-Co _3_ O _4_ , Co-Co _3_ O _4_ , Pd-Co _3_ O _4_ , and Pt-Co _3_ O _4_ catalysts, respectively. The (111) planes at around 2ϴ value of 45° were selected to calculate the grain sizes of Ni and Co catalysts. Similarly, the (111) planes at around 2ϴ value of 40° were selected to calculate the grain size of Pd and Pt catalysts.

**Figure 2:**
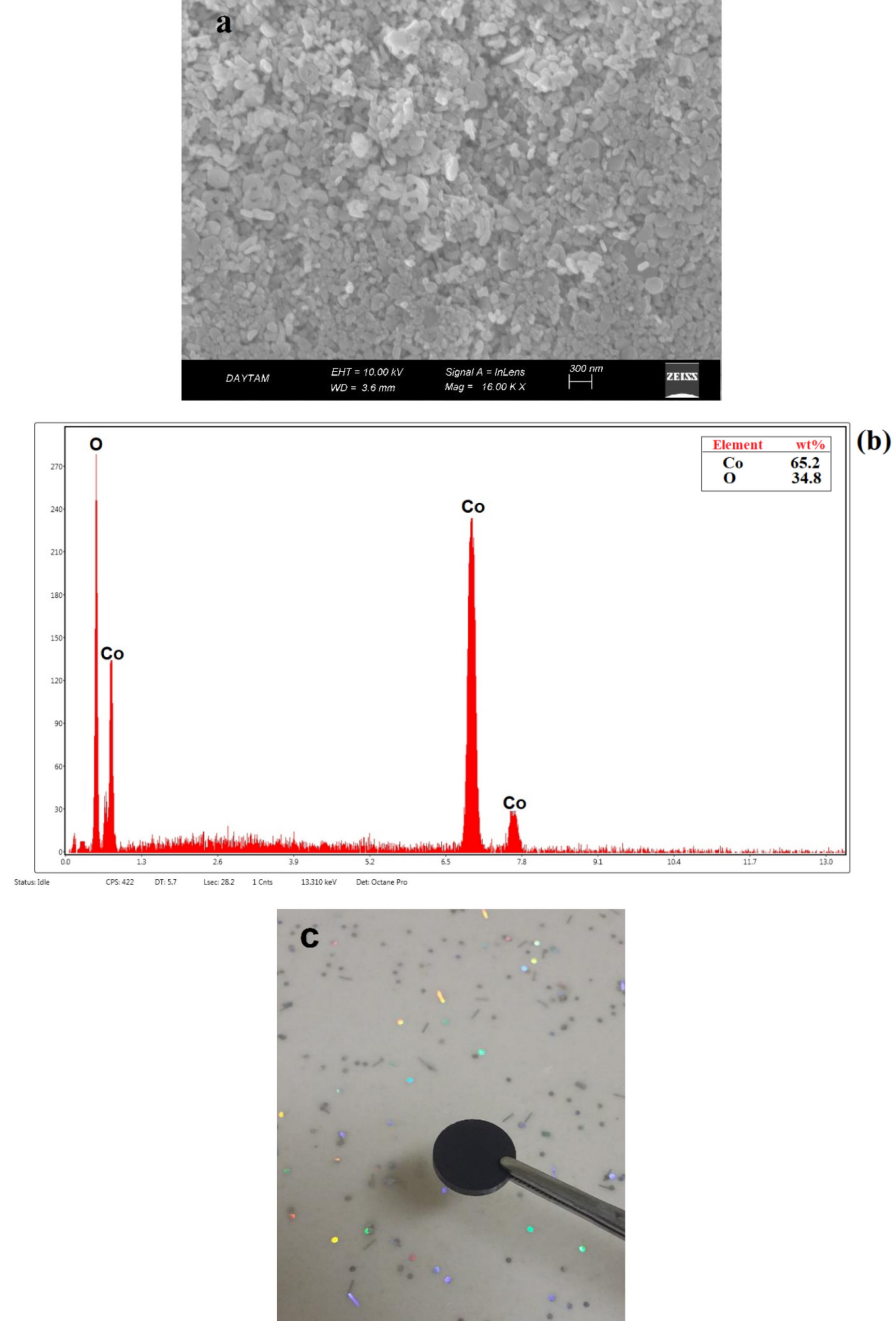
a) SEM image b) EDS analysis c) photo for Co _3_ O _4_ pellet.

The SEM image given in Figure 2a shows surface morphologies (particle nature) for the Co _3_ O _4_ support material in pellet form. Particle formation with homogeneous dispersion was observed in Co _3_ O _4_ support material, according to the EDS analysis given for Co _3_ O _4_ in Figure 2b. In addition, Figure 2c shows the prepared Co _3_ O _4_ pellet.

**Figure 3:**
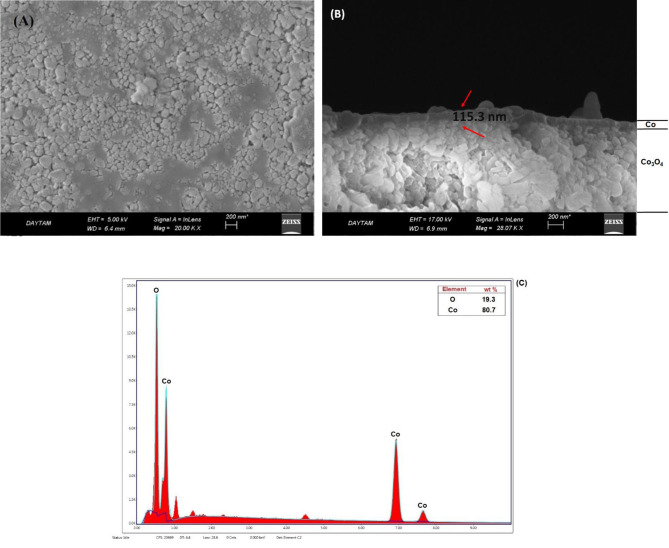
A) Surface B) cross sectional SEM images, and C) EDS analysis result for Co-Co _3_ O _4_ catalyst.

**Figure 4:**
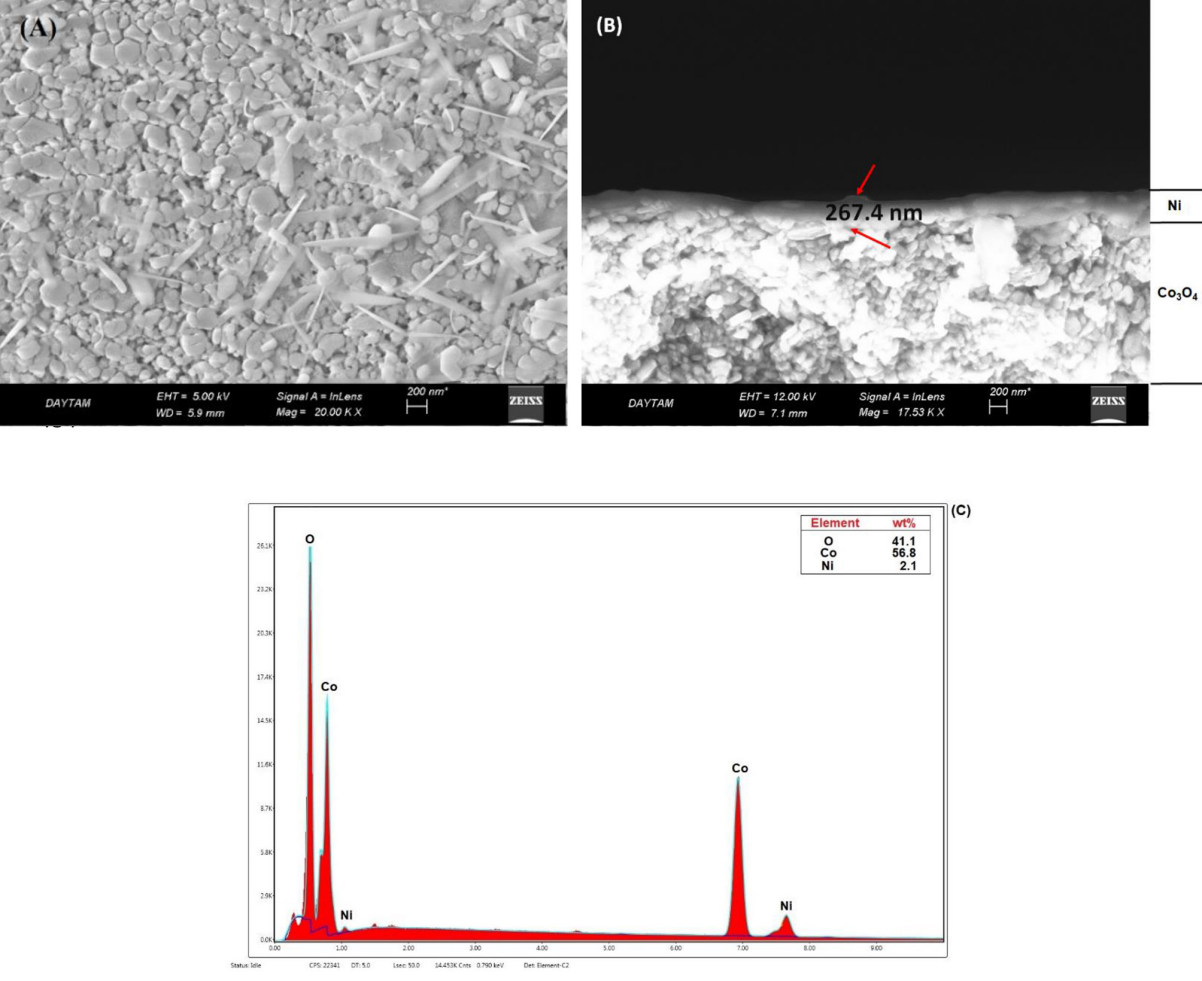
A) Surface B) cross sectional SEM images, and C) EDS analysis result for Ni-Co _3_ O _4_ catalyst.

**Figure 5:**
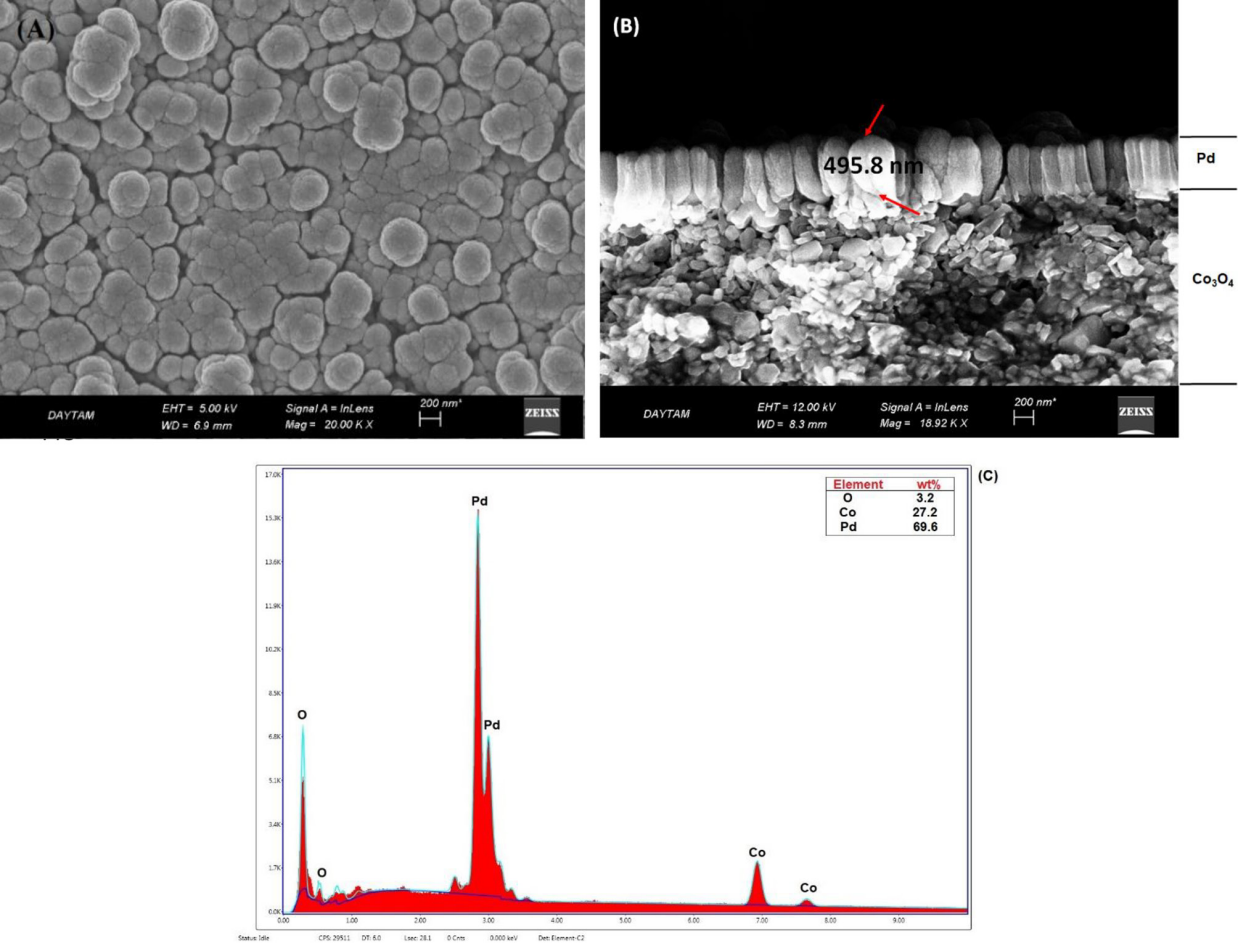
A)Surface B) cross sectional SEM images, and C) EDS analysis result for Pd-Co _3_ O _4_ catalyst.

**Figure 6:**
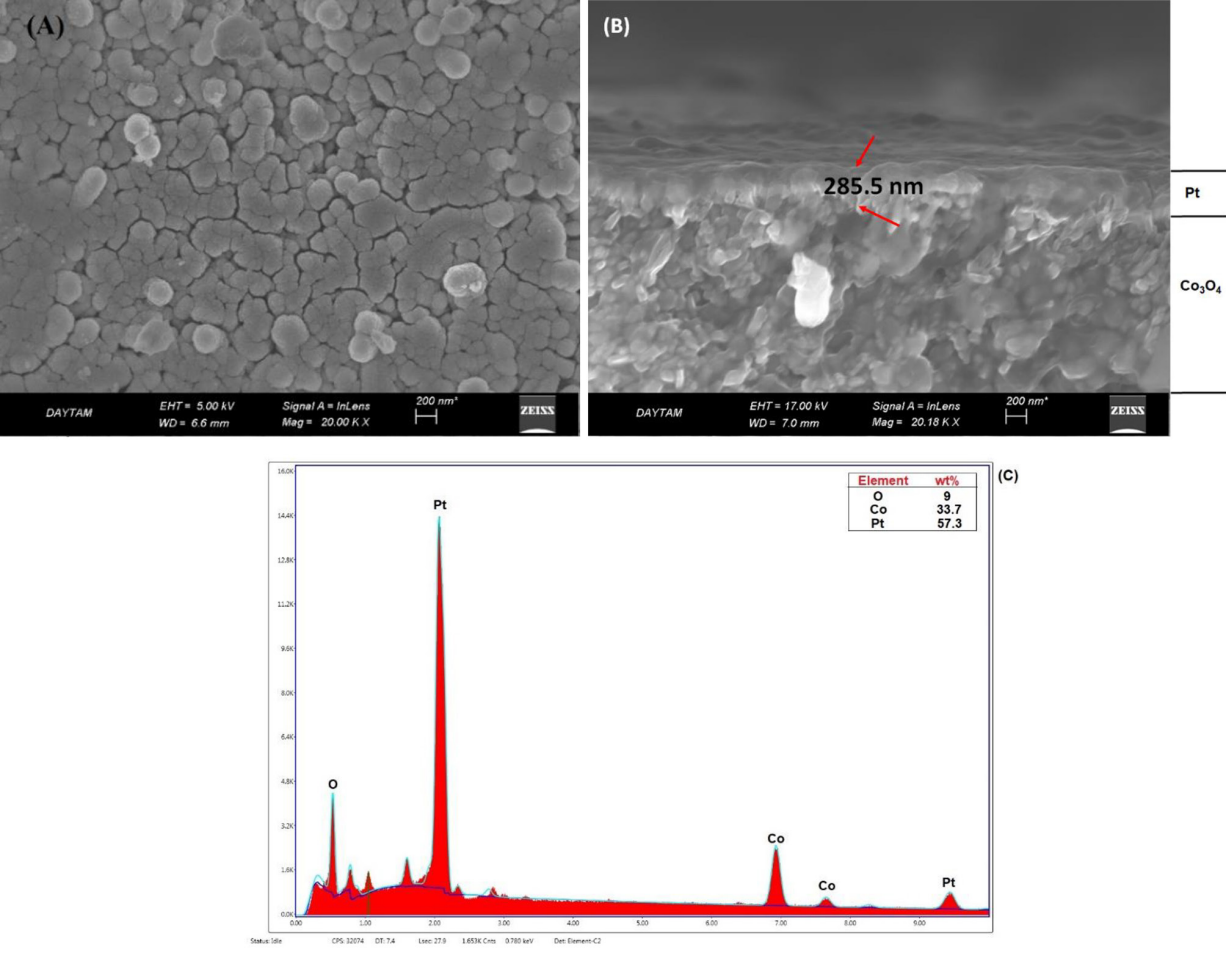
A) Surface B) cross sectional SEM images, and C) EDS analysis result for Pt-Co _3_ O _4_ catalyst.

Figures 3–6 show SEM images of prepared catalysts from both the surface and cross sectional areas as well as the EDS results for the catalysts.

Figures 3a–c show the surface and cross sectional SEM images and EDS analysis of Co-Co _3_ O _4_ . According to Figure 3a, particle formation was observed with nonhomogeneous dispersion for Co-Co _3_ O _4_ . The Co layer thickness was clearly observed from the cross sectional SEM images of the catalyst (Figure 3b). Catalyst thickness was approximately 115.3 nm. According to the EDS spectrum (Figure 3c), Co and O elements were detected for the catalyst.

Figures 4a–c show the surface and cross sectional SEM images and EDS analysis of Ni-Co _3_ O _4_ . According to Figure 4a, particle formation was observed with nonhomogeneous dispersion for Ni-Co _3_ O _4_ . The Ni layer thickness was clearly observed from the cross sectional SEM images of the catalyst (Figure 4b). Catalyst thickness was approximately 267.4 nm. According to the EDS spectrum(Figure4c) Ni, Co, and O elements were detected for the catalyst. A low nickel-coating ratio was observed in this case.

Figures 5a–c show the surface and cross sectional SEM images and EDS analysis of Pd-Co _3_ O _4_ .According to Figure5a, particle formation had homogeneous dispersion compared to Co- and Ni-based catalysts for Pd-Co _3_ O _4_ . In addition, the Pd particle sizes were larger than Ni and Co particles. This confirms the average particle size results calculated for the catalysts using XRD data. The Pd layer thickness was clearly observed from cross sectional SEM images of the catalyst (Figure 5b). Catalyst thickness was approximately 495.8 nm. According to the EDS spectrum (Figure 5c) Pd, Co, and O elements were detected for the catalyst, and a severe peak of Pd was observed.

Figures 6a–c show the surface and cross sectional SEM images and EDS analysis of Pt-Co _3_ O _4_ . Homogeneous particle formation was observed for Pt-Co _3_ O _4_ catalyst, similar to the Pd-Co _3_ O _4_ catalyst (Figure 6a). The Pt layer thickness was clearly observed from the cross sectional SEM images of the catalyst (Figure 6b). Catalyst thickness was approximately 285.5 nm. According to the EDS spectrum (Figure 6c), Pt, Co, and O elements were detected for the catalyst, and a severe peak of Pt was observed.

**Figure 7:**
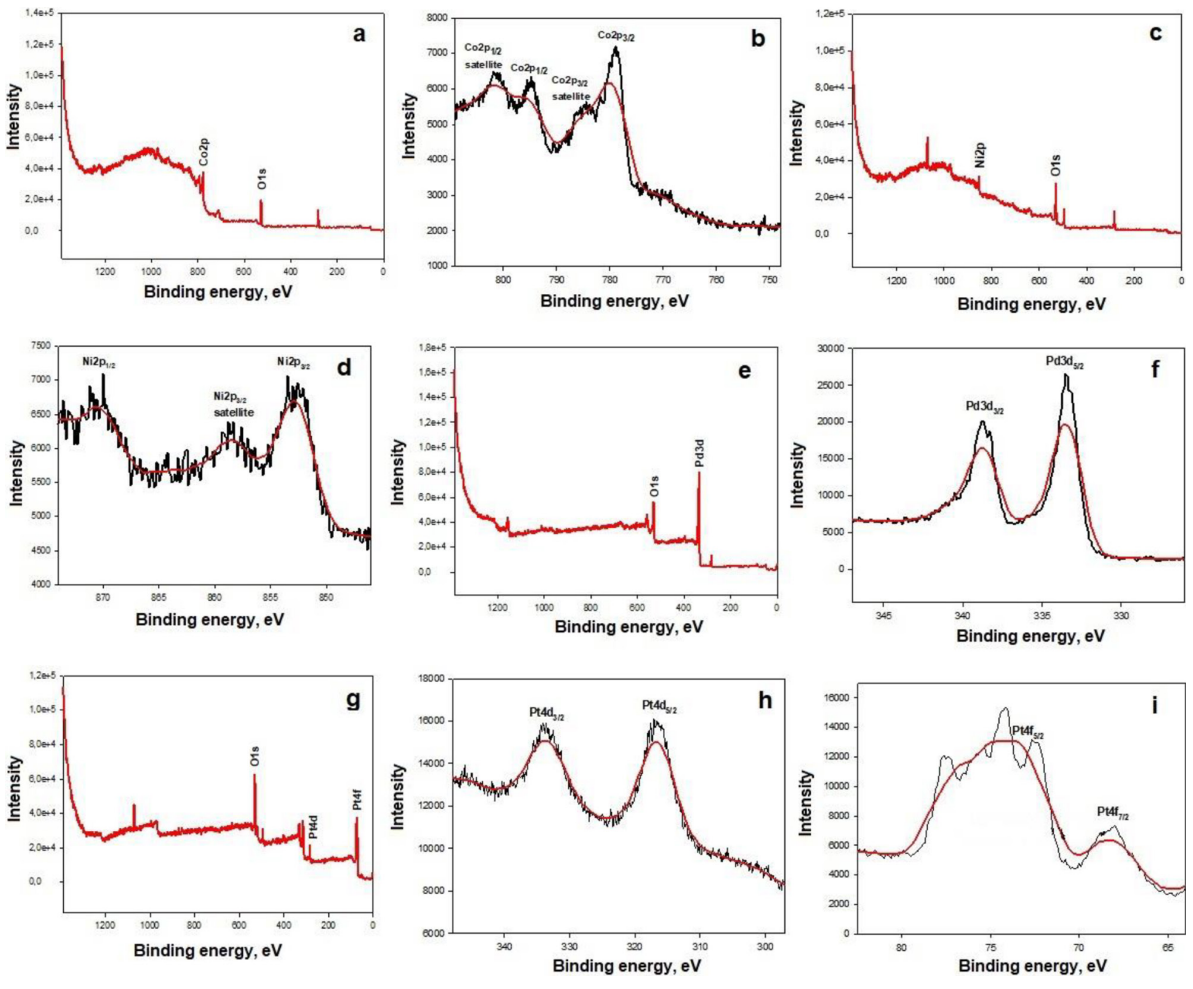
XPS spectrums for; Co-Co _3_ O _4_ catalyst (a) general spectrum (b) Co 2p, Ni- Co _3_ O _4_ catalyst (c) general spectrum (d) Ni 2p, Pd- Co _3_ O _4_ catalyst (e) general spectrum (f) Pd 3, Pt- Co _3_ O _4_ catalyst (g) general spectrum (h) Pt 4d (i) Pt 4f electron regions.

Figure 7 illustrates the XPS spectra of general and Co 2p, Ni 2p, Pd 3d, and Pt 4d–4f level photoemission signals of the catalysts. Figures 7a, 7c, 7e, and 7g show the XPS spectra of general elements. According to Figure 7b, two prominent peaks were observed for Co 2p _3/2_ and Co 2p _1/2_ (779.6 eV and 795.5 eV, respectively). Two shake-up satellites at 802.8 and 787.2 eV indicate the presence of Co _3_ O _4_ [24]. Two peaks of 2p _3/2_ and 2p _1/2_ for Ni were observed at 852.7 and 870.6 eV, respectively (Figure 7d) [25]. Figure 7f shows 334.5 eV(3d _5/2_ ) and 338.8 eV(3d _3/2_ ) corresponding to the Pd metal and Pd ^+2^ states, respectively [26]. The peaks located at binding energies of 316.3 and 333.2 eV (Figure 7h) can be attributed to the Pt 4d _5/2_ and Pt 4d _3/2_ regions, respectively [27].Furthermore, Figure 7i shows Pt 4f _7/2_ and Pt 4f _5/2_ peaks, demonstrating the reduction of Pt(IV) to Pt(0) [28].

### 3.2. Hydrogen generation of the catalysts

**Figure 8:**
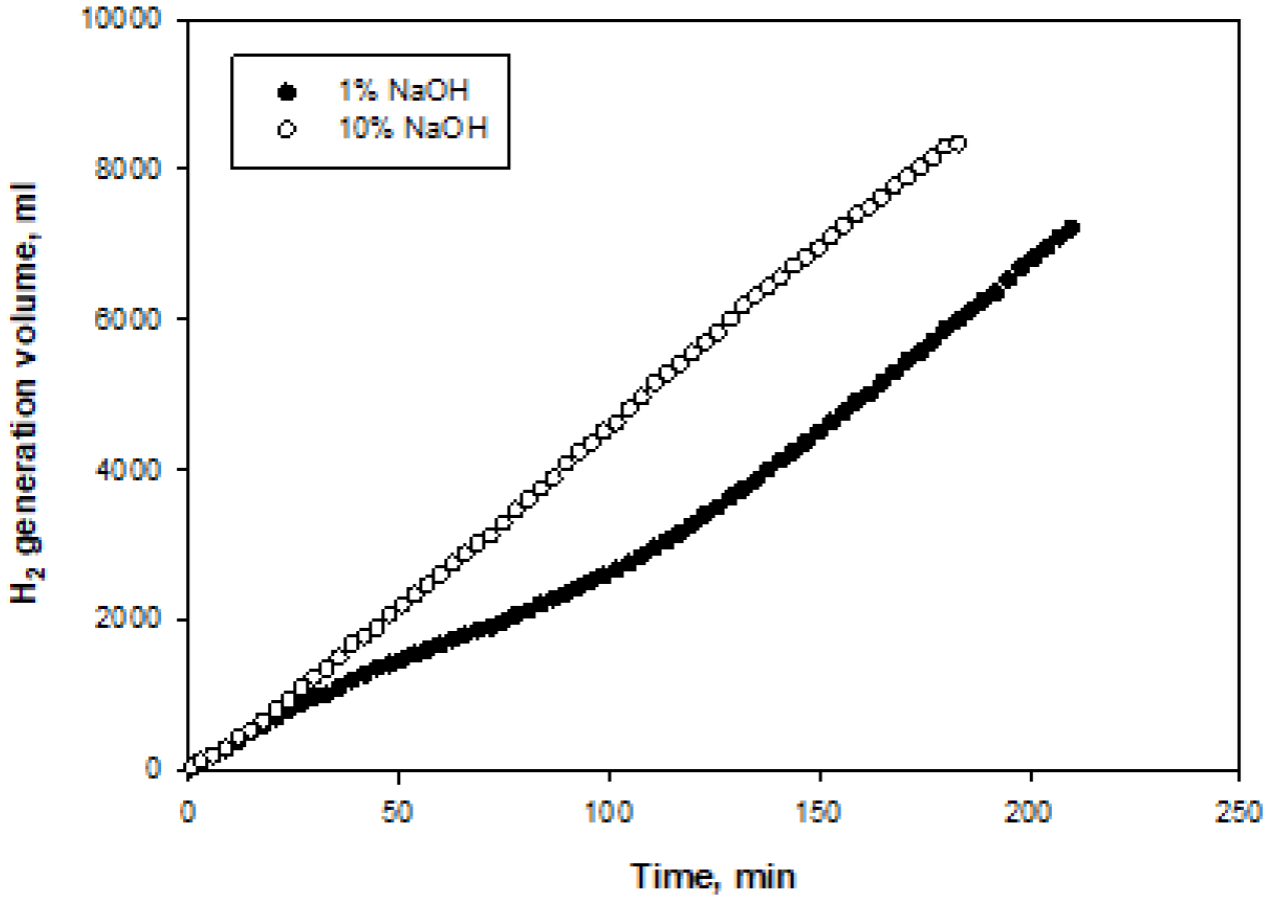
Time-dependent volumes of hydrogen generated at two different NaOH initial concentrations for Co-Co _3_ O _4_ catalyst.

**Figure 9:**
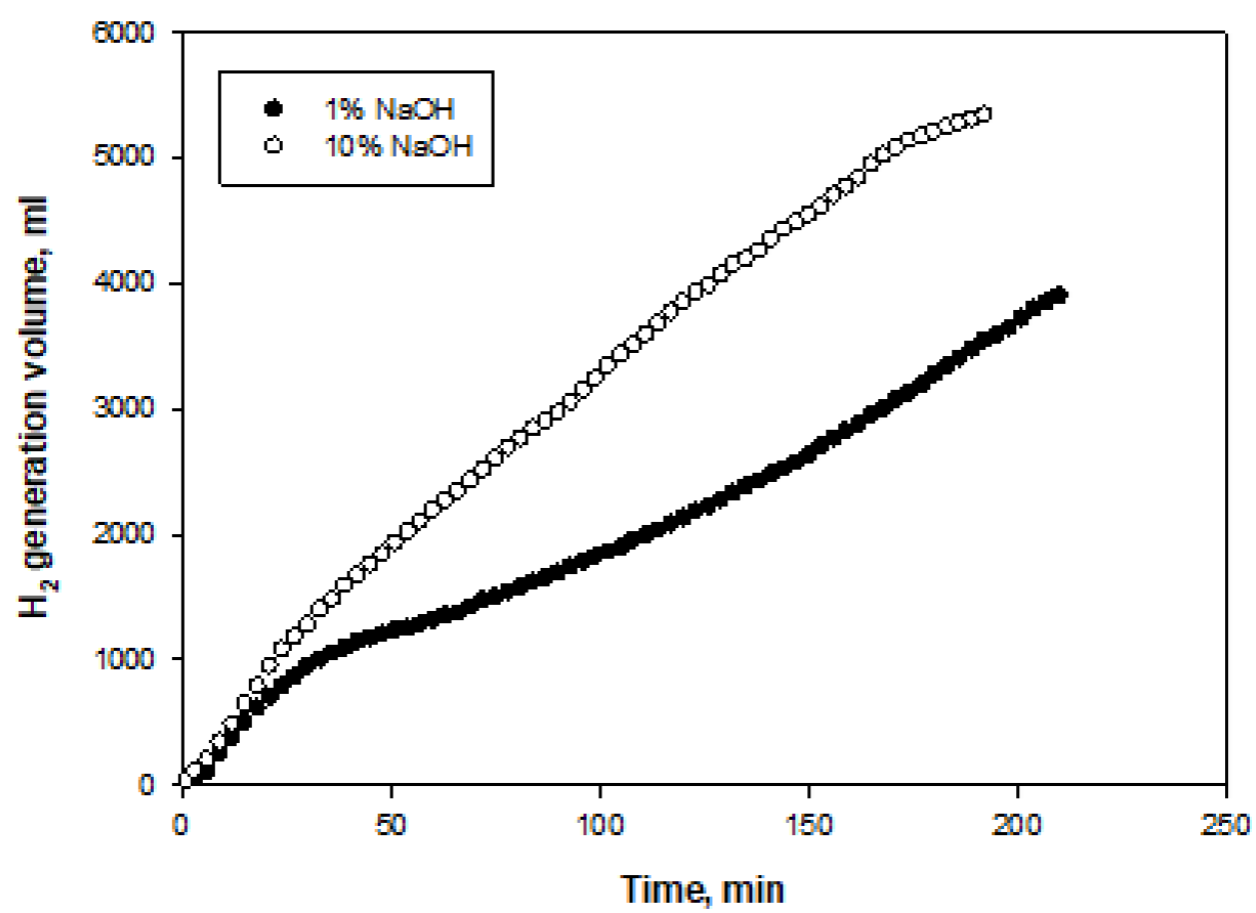
Time-dependent volumes of hydrogen generated at two different NaOH initial concentrations for Ni-Co _3_ O _4_ catalyst.

**Figure 10:**
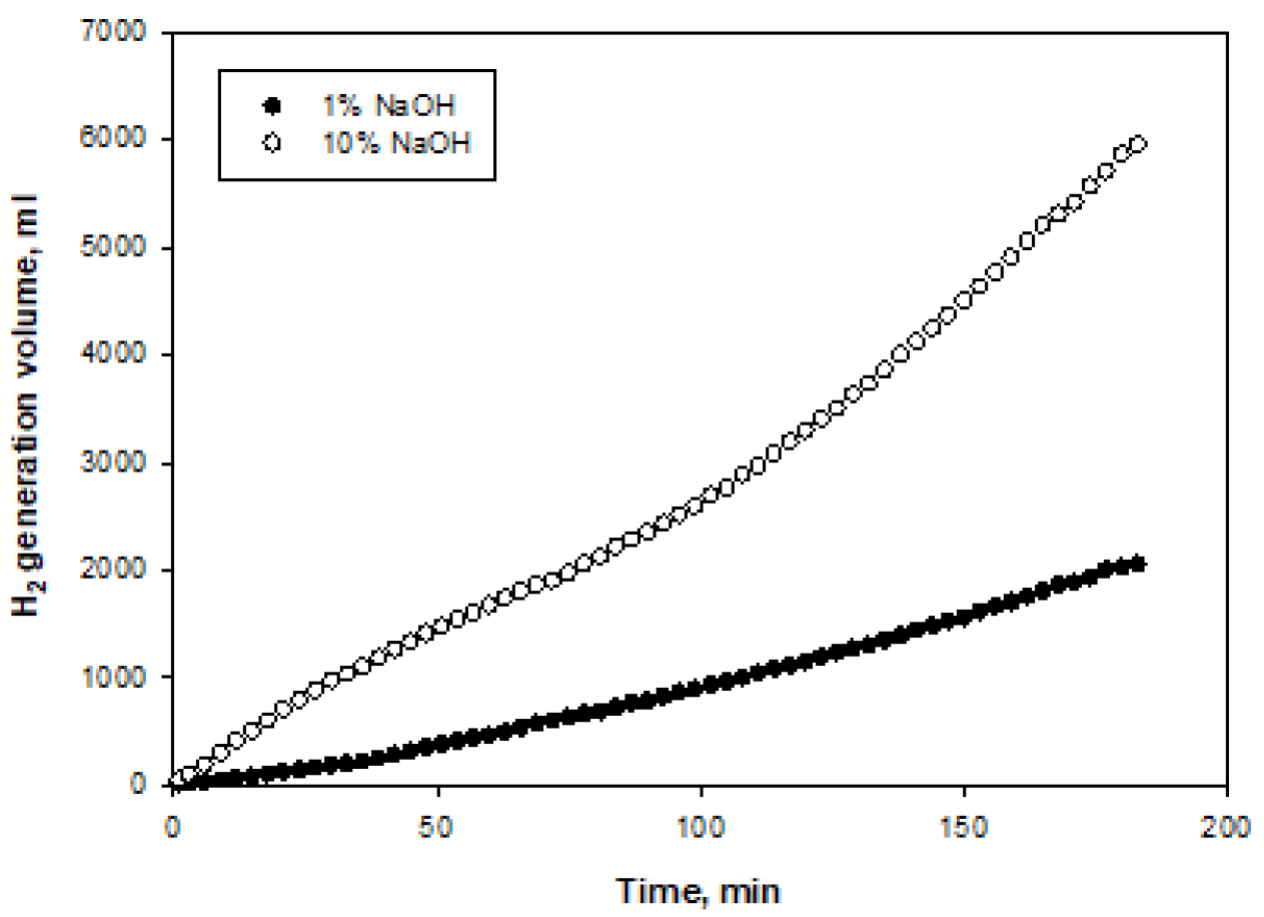
Time-dependent volumes of hydrogen generated at two different NaOH initial concentrations for Pd-Co _3_ O _4_ catalyst.

**Figure 11:**
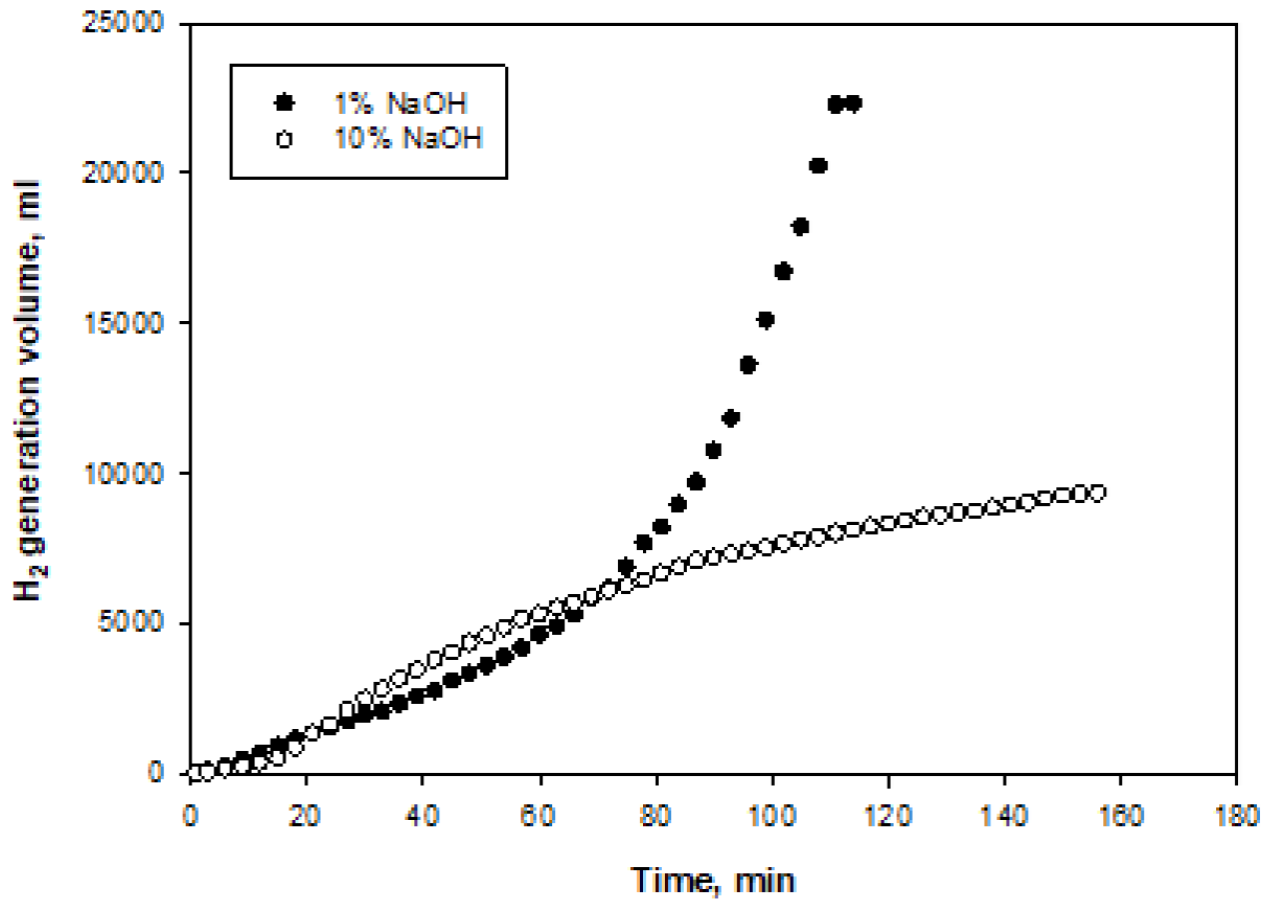
Time-dependent volumes of hydrogen generated at two different NaOH initial concentrations for Pt-Co _3_ O _4_ catalyst.

Figures 8–11 show the amounts of hydrogen generated from NaBH _4_ hydrolysis at 1 wt% and 10 wt% NaOH initial concentrations. All the experiments were performed at 25°C and 10 wt% NaBH _4_ . The hydrogen generation rates of the catalysts for 1% and 10%NaOH initial concentrations are given in Table 2. Increasing the initial NaOH concentration for Ni, Co, and Pd-Co _3_ O _4_ catalysts caused an increase in the hydrogen generation rate (Figures 8–10). This increase was more than 2.5 times for the Pd-Co _3_ O _4_ catalyst. An increase in the hydrogen generation rate of the Pd-based catalyst following an increase in the NaOH initial concentration was observed in a previous study by our group and was attributed to inclusion of the hydroxyl ion inNaBH _4_ hydrolysis [21]. Similarly, the increase in NaOH initial concentration for Ni- and Co-based catalysts had a positive effect [22].The Co-Co _3_ O _4_ catalyst was the most active among the three catalysts. The hydrogen generation rate for the Co-Co _3_ O _4_ catalyst at an initial concentration of 10% NaOH was 945 mL/g _cat_ .min. InRakap et al. the activity of a Co-Ni-P/Pd-TiO _2_ catalyst prepared with an electroplating method was investigated at 25°C, and the hydrogen generation rate was 460 mL/g _cat_ .min [29]. Similarly, in a study by Krishnan et al., the hydrogen generation rate of a Co-B catalyst prepared on Ni foam was 300 mL/g _cat_ .min [30]. In contrast, when the initial concentration of NaOH for the Pt catalyst increased from 1% to 10%, a decrease in the hydrogen generation rate was observed (Figure 11). The highest hydrogen generation rate was obtained from the Pt-Co _3_ O _4_ catalyst in 1% NaOH initial concentration (1653 mL/g _cat_ .min). At an initial concentration of 10% NaOH in the Pt-Co _3_ O _4_ catalyst, the hydrogen generation rate decreased due to the reduced amount of free water required for the reaction and the low solubility of the reaction by-product NaBO _2_ [21]. Hydrogen generation rates from the NaBH _4_ hydrolysis of Ni-, Co-, Pd-, and Pt-based catalysts prepared in this work, and catalysts prepared using different thin film methods described in the literature, were compared in Table 3 [12–17, 29–33]. As shown in Table 3, hydrogen generation rates changed significantly depending on the catalysts used as well as the thin film preparation techniques. In this study, the efficiency of thin film catalysts prepared by the DC magnetron sputtering method for noble and nonnoble metals was investigated, and promising results were observed.

**Table 2:** H2 generation rate (HGR) of catalysts at two different NaOH initial concentrations.

Catalyst	HGR, ml/g _cat_ .min	
	1% NaOH	10% NaOH
Co-Co _3_ O _4_	587	945
Ni-Co _3_ O _4_	568	782
Pd-Co _3_ O _4_	229	614
Pt-Co _3_ O _4_	1653	1382

**Table 3:** HGRs for hydrolysis of NaBH _4_ catalyzed by various catalysts in the literature.

Catalysts	Method	Operating conditions			HGR, ml/g _cat_ .min	Film thickness, nm	Ref
		wt% NaBH _4_	wt% NaOH	Temp. (oC)			
Co-Ni-P/Pd-TiO _2_	electroless plating	0.3 M	10	25	460	-------	[29]
Co-B/Ni foam	electroplatingelectroless plating	10	5	25	300 1640	10000-15000	[14]
Co-P/Cu	electroplating	10	1	30	954	Increase with an increase in duration	[30]
Co film/Cu foil	magnetic-field-induced chemical reduction	0.2 M	0.4 M	25	1270	-------	[16]
Co-P/Cu sheet	electroless plating	5	1	30	2275.1	-------	[15]
Co/Ni foam	magnetron sputtered	1M	4.5	25	2650	2000	[12]
Co–B/Pd	dry dip-coating	20	1M	30	2875	-------	[17]
Co-B/ silicon	PLD	1	5	room	3300	Particle sizes (180-300 nm)	[31]
Co–Ni–P/Cu	electroplating	10	10	25	3636	-------	[32]
							
Co–P–B	PLD	0.025 M	0.025 M	room	4230	-------	[13]
							
Co-W-P/Cu	electroplating	10	10	30	5000	-------	[33]
Pd-Co _3_ O _4_ Ni-Co _3_ O _4_ Co-Co _3_ O _4_	DC magnetron sputtered	10	10	25	614 782 945	495.8 267.4 115.3	This work
Pt-Co _3_ O _4_	DC magnetron sputtered	10	1	25	1653	285.5	This work
PLD: Pulsed laser deposition DC: direct current							

## 4. Conclusions

In this study, Ni, Co, Pd, and Pt metals supported by a Co _3_ O _4_ pellet were prepared using a DC magnetron sputtering method for hydrogen generation from the hydrolysis of NaBH _4_ . The SEM images for the catalysts illustrated surface morphologies and cross sectional areas. The Pd and Pt particles have nearly uniform size, and good dispersions were obtained. Catalyst layer thicknesses were clearly observed at 115.3, 267.4, 495.8, and 285.5 nm for Co, Ni, Pd, and Pt, respectively. According to the XRD results, the highest particle size obtained from a Pd-based catalyst was approximately 33.9 nm. The hydrogen generation rates of the catalysts were investigated at 1% and 10% NaOH initial concentrations. An increase in the NaOH initial concentration provides an increase in the rate of hydrogen generation for Co, Ni, and Pd catalysts. The minimum hydrogen generation rates were observed with a Pd-based catalyst. The reason may be that the Pd-based catalyst has a higher average particle size and a higher catalyst thickness than other catalysts. The highest hydrogen generation rate was obtained from the Pt-Co _3_ O _4_ catalyst in 1% NaOH initial concentration (1653 mL/g _cat_ .min).
